# DNA assays for genetic discrimination of three *Phragmites australis* subspecies in the United States

**DOI:** 10.1002/aps3.11512

**Published:** 2023-03-08

**Authors:** Denise L. Lindsay, Xin Guan, Nathan E. Harms, James T. Cronin, Laura A. Meyerson, Richard F. Lance

**Affiliations:** ^1^ United States Army Engineer Research and Development Center Environmental Laboratory Vicksburg Mississippi 39180 USA; ^2^ Bennett Aerospace Vicksburg Mississippi 39180 USA; ^3^ Louisiana State University Baton Rouge Louisiana 70803 USA; ^4^ University of Rhode Island Kingston Rhode Island 02881 USA; ^5^ Present address: ModernaTX, Moderna Technology Center Norwood Maryland USA

**Keywords:** aquatic invasive species, chloroplast genome sequencing, environmental genetics, hydrolysis probe real‐time quantitative PCR, plant identification

## Abstract

**Premise:**

To genetically discriminate subspecies of the common reed (*Phragmites australis*), we developed real‐time quantitative (qPCR) assays for identifying *P. australis* subsp. *americanus*, *P. australis* subsp. *australis*, and *P. australis* subsp. *berlandieri*.

**Methods and Results:**

Utilizing study‐generated chloroplast DNA sequences, we developed three novel qPCR assays. Assays were verified on individuals of each subspecies and against two non‐target species, *Arundo donax* and *Phalaris arundinacea*. One assay amplifies only *P. australis* subsp. *americanus*, one amplifies *P. australis* subsp. *australis* and/or *P. australis* subsp. *berlandieri*, and one amplifies *P. australis* subsp. *americanus* and/or *P. australis* subsp. *australis*. This protocol enhances currently available rapid identification methods by providing genetic discrimination of all three subspecies.

**Conclusions:**

The newly developed assays were validated using *P. australis* samples from across the United States. Application of these assays outside of this geographic range should be preceded by additional testing.

Convergent evolution, ecophenotypic variation, age‐related morphological changes, hybridization, and the occurrence of cryptic species are some of the potentially confounding factors that make identifying some plants morphologically with absolute confidence difficult. This is especially true when populations include native and introduced lineages of the same species or subspecies (Meyerson et al., 2012; Gabby, 2020). The common reed, *Phragmites australis* (Cav.) Trin. ex Steud., is a perennial grass found in wetlands, marshes, river edges, lake shores, ponds, and roadside ditches across North America, tolerating fresh to mesohaline salinities (Lambert et al., 2010). Three subspecies of *P. australis* co‐occur in North America (Saltonstall, 2016): one native (*P. australis* subsp. *americanus* Saltonst., P. M. Peterson & Soreng), one introduced (*P. australis* subsp. *australis*), and one with uncertain origins (*P. australis* subsp. *berlandieri* (E. Fourn.) Saltonst. & Hauber).

*Phragmites australis* subsp. *americanus* is widespread across the United States, excluding the southeastern states (Saltonstall et al., 2004). *Phragmites australis* subsp. *australis* is of European origin, with multiple introductions leading to its spread across North America during the past couple of centuries (Saltonstall, 2002; Kirk et al., 2011); it is now recorded in every U.S. state (Saltonstall et al., 2004). *Phragmites australis* subsp. *berlandieri* is found along the U.S. Gulf Coast (Saltonstall, 2016). While cryptogenic in the United States (Lambert et al., 2016), with many populations being considered hybrids of *P. australis* subsp. *berlandieri* and *P. mauritianus* Kunth (Lambertini et al., 2012; Lambert et al., 2016) or *P. karka* (Retz.) Trin. ex Steud. (Saltonstall and Hauber, 2007), it is not considered invasive in most areas (Lambert et al., 2010). Commonly referred to as the Gulf Coast common reed, it is not endemic to the United States, but is native to Mexico, where multiple new haplotypes have been discovered (Colin and Eguiarte, 2016), and likely spread northward into the United States during the Pleistocene (Colin and Eguiarte, 2016). Both the introduced and Gulf Coast common reed are exhibiting range expansion, with more recent establishments in the U.S. Southwest, the only region worldwide that is inhabited by all three subspecies (Meyerson et al., 2010).

Native populations of common reed are ecologically important for maintaining high‐quality wildlife habitat and species diversity, and are legally protected in many locations (Martin and Blossey, 2013); however, *P. australis* subsp. *americanus* is often outcompeted by the introduced subspecies, which grows more aggressively (Gabby, 2020) and can form dense monotypic stands (Swearingen and Saltonstall, 2010). *Phragmites australis* subsp. *australis* is managed by mowing, flooding, burning, and herbicide application because it alters wetland hydrology (increasing evaporation and trapping sediment) and degrades wildlife habitat, often reducing biodiversity (Chambers et al., 1999; Tewksbury et al., 2002). In many cases, genetic discrimination between the native and introduced common reed is important to natural resource managers interested in controlling invasions of the exotic subspecies while conserving populations of the native subspecies.

Although negative ecological impacts have been documented across the United States as resulting from the introduced subspecies, both *P. australis* subsp. *australis* and *P. australis* subsp. *berlandieri* provide important ecological services along the northern coast of the Gulf of Mexico. Their thick networks of roots/rhizomes provide storm surge protection and reduce erosion of coastal lands (Meyerson et al., 2009; Saleh and Weinstein, 2016). Whereas these subspecies thrive in conditions on the northern Gulf Coast (e.g., high salinity and moderate water depth), the native subspecies is absent (Saltonstall et al., 2005). However, Gulf Coast *Phragmites* stands are being negatively impacted by a nonindigenous scale insect (*Nipponaclerda biwakoensis*), with apparent differences in damage levels among *Phragmite*s lineages in the region (Knight et al., 2018; Cronin et al., 2020). Chromosome ploidy level may also play a role in plant response to the scale insect (Meyerson et al., 2020), as *P. australis* subsp. *australis* is tetraploid and *P. australis* subsp. *berlandieri* is both tetraploid and hexaploid (Meyerson et al., 2016). Discriminating among subspecies thus becomes particularly important to coastal vegetation management, erosion, and flood control in the region. Additionally, in other regions of the United States, subspecies discrimination may be key to effective biocontrol of the introduced common reed (Tewksbury et al., 2002). As such, all three subspecies are mapped and managed throughout much of the United States and Canada.

To assist with mapping and management, and to streamline the genetic discrimination of *P. australis*, we created a tool capable of quickly identifying all three subspecies present in the United States by designing a set of novel DNA assays. The DNA assays developed here are real‐time quantitative PCR (qPCR) assays targeting DNA loci unique to a particular taxon, such that they may be used to clearly identify specimens of that taxon. DNA assays have been shown to be a reliable tool to discriminate between morphologically similar plants (Duminil and Michele, 2009). The assays described in this study are hydrolysis probe–based qPCRs, capable of discriminating across very small differences in DNA sequence (1–3 base pairs) in the target loci. These assays are highly effective and provide more rapid differentiation of unidentified samples than other currently available *Phragmites* genetic discrimination methods (e.g., sequencing, Saltonstall, 2002; genotyping, Saltonstall, 2003; RNase H–dependent PCR, Zuzak et al., 2018) and can differentiate all three subspecies, unlike some *Phragmites* genetic discrimination methods (e.g., restriction fragment length polymorphism [PCR‐RFLP], Wendell et al., 2021). Our hope is that further work and technological advances will allow these assays to be easily utilized for in‐field plant identification.

## METHODS AND RESULTS

Plant tissue samples (leaves) were collected from individual *P. australis* plants (*n* = 40) maintained at Louisiana State University, Baton Rouge, Louisiana, and included individuals representative of the different subspecies and lineages found across the United States. Additional samples were obtained from a U.S. Geological Survey greenhouse maintained at the Great Lakes Science Center in Ann Arbor, Michigan (*n* = 8); a University of Rhode Island greenhouse in Kingston, Rhode Island (*n* = 7); and field collections from Ohio (*n* = 1) and New York (*n* = 3) (Appendix 1). Leaf tissue samples from two morphologically similar, non‐target confamilial species that co‐occur with *Phragmites* in the study region, *Arundo donax* L. (*n* = 4) and *Phalaris arundinacea* L. (*n* = 4), were also obtained from the field for cross‐amplification tests.

DNA extractions using a modified cetyltrimethylammonium bromide (CTAB) method (Doyle and Doyle, 1987; Lalhmangaihi et al., 2015) were performed for all samples (*N* = 67; Appendix 1) after an initial disruption step with a bead beater and Lysing Matrix D tubes (MP Biomedicals, Irvine, California, USA). Chloroplast DNA extractions were performed for nine of those samples, including all three subspecies and five biotypes (Table 1), following the Mariac et al. (2000) protocol. Genomic DNA concentrations (ng/µL) were determined with the NanoDrop One/One UV‐Vis Spectrophotometer (NanoDrop Technologies, Wilmington, Delaware, USA). DNA used for sequencing and primer testing had purity values (260/280 ratio) ranging from 1.77–1.98, and all samples were normalized to 1 ng/µL for qPCR assays.

**Table 1:** Chloroplast sequence sample information, including region of the United States, lineage, *Phragmites australis* subspecies, biotype as described in Lambertini et al. (2012) and Saltonstall (2016), collection state and location (latitude and longitude), and NCBI GenBank accession number.

Region	Lineage	*P. australis* subspecies	Biotype	Collection location			Accession number
				State	Latitude	Longitude	
Gulf Coast	Introduced	*australis*	Greeny	Louisiana	29.21941	−89.30135	MW287627
			Delta	Louisiana	29.23722	−89.38607	MW287628
	Gulf Coast	*berlandieri*	Land	Louisiana	29.23722	−89.38607	MW287629
Great Lakes	Introduced	*australis*	European	Wisconsin	42.47901	−87.84930	MW287630
			European	Michigan	44.29368	−85.24337	MW287633
	Native	*americanus*	Native	Minnesota	46.74054	−92.06205	MW287631
			Native	Missouri	40.06046	−95.24317	MW287632
			Native	Michigan	42.33793	−84.00016	MW287634
			Native	Ohio	41.67585	−83.30478	MW287635

Native (*P. australis* subsp. *americanus*: *n* = 4), introduced (*P. australis* subsp. *australis*: *n* = 4), and Gulf Coast (*P. australis* subsp. *berlandieri*: *n* = 1) samples representing all U.S. lineages, and as many recognized “biotypes” within our study regions as were available in our sample pool, were selected for chloroplast sequencing (Table 1; Lambertini et al., 2012; Saltonstall, 2016). Each sample was prepared according to the protocol outlined for the Nextera DNA Flex Library Prep Kits (Illumina, San Diego, California, USA) for chloroplast whole genome sequencing. Sequencing was performed on the Illumina MiSeq system (Illumina) using the 600‐cycle MiSeq Reagent V3 kit (Illumina). MiSeq Reporter Software (Illumina) was used to sort the pool of sequences by indices to identify the sequences from each sample. Chloroplast DNA genomes were assembled by aligning the reads of a reference sequence from the National Center for Biotechnology Information (NCBI) GenBank genetic sequence database (KJ825856.1) using the medium sensitivity/fast settings in Geneious Prime software (Biomatters Ltd., Auckland, New Zealand). Consensus sequences from each sample's whole chloroplast DNA were extracted and then aligned using global alignment with the free end gaps type function in Geneious Prime 2019.2.3. The entire chloroplast sequences (>137,500 bp) for each of the nine samples (Table 1) were submitted to the NCBI GenBank genetic sequence database; assigned accession numbers included: *P. australis* subsp. *americanus* (MW287631, MW287632, MW287634, MW287635), *P. australis* subsp. *australis* (MW287627, MW287628, MW287630, MW287633), and *P. australis* subsp. *berlandieri* (MW287629).

These chloroplast sequences were then used to identify multiple DNA loci with putative subspecies‐unique sequence, from which nine qPCR assays (e.g., forward and reverse primers, and internal hydrolysis probe) were designed using Geneious and Primer3 (Rozen and Skaletsky, 2000). These assays were tested for expected detection/non‐detection (i.e., detectable PCR amplification) in the nine original *Phragmites* samples used for chloroplast genome sequencing (chloroplast DNA extractions), of which three assays (one amplifying samples from *P. australis* subsp. *americanus* [AMER], one amplifying samples of *P. australis* subsp. *australis* and/or *P. australis* subsp. *berlandieri* [AUBE], and one amplifying samples of *P. australis* subsp. *americanus* and/or *P. australis* subsp. *australis* [AMAU]) (Table 2) performed as expected and went through further optimization. Optimization of the three assays included minor adjustments to annealing temperature and cycle number in order to maximize amplification while maintaining non‐amplification of non‐target species/subspecies. These three optimized assays were then validated using CTAB DNA extractions in all collected samples from each of the three subspecies (*P. australis* subsp. *americanus*: *n* = 24, *P. australis* subsp. *australis*: *n* = 26, and *P. australis* subsp. *berlandieri*: *n* = 9, including *n* = 9 “blind” samples for which only our collectors knew the morphological identity). The assays were then verified against four samples from each of the two non‐target species, *A. donax* and *P. arundinacea*. We determined detection/non‐detection results and threshold cycle (Ct) values over three technical replicates of each sample.

**Table 2:** Chloroplast DNA assays, including qPCR primers and probes, designed and tested for *Phragmites australis* subspecies, listing assay name, primer or probe designation, primer or probe sequence, amplicon length, gene of origin, and targeted subspecies.

Assay	Primer or probe	Primer or probe sequence	Amplicon length (bp)	Gene of origin	Target *P. australis* subsp.
AMER	AMER‐F	AAAAACTAAGAGATGGGTGAAA	81	trnT–trnL spacer + trnL regions	*americanus*
	AMER‐P	ACAAGTACACAAGGAATCCTGGT			
	AMER‐R	TCGCCATATCCCCATTTTCCTT			
AMAU	AMAU‐F	AAGACCGTCCTGATATATTAAGTAGG	81	psaA‐ORF170 intergenic spacer	*americanus* + *australis*
	AMAU‐P	AGATTGCCCCTTTTATTTGCTTT			
	AMAU‐R	GGATAGGCTCTAGAACAGAAGT			
AUBE	AUBE‐F	AAAAACTAAGAGATGGGTGA	72	trnT–trnL intergenic spacer region	*australis* + *berlandieri*
	AUBE‐P	AAATTACACAAGGAATCCTGGT			
	AUBE‐R	TCGCCATATCCCCATTTTCCTT			

All qPCR reactions were run in 20 µL volumes containing 1X TaqMan Environmental Master Mix 2.0 (Thermo Fisher Scientific, Waltham, Massachusetts, USA), 0.5 µM of each primer, 0.125 µM of each probe, and 1 µL of DNA template (1 ng/µL). Temperature cycling began with an initial denaturation step at 95°C for 10 min, followed by 40 cycles of 95°C for 15 s and 60°C for 1 min. qPCR reactions were run on an Applied Biosystems ViiA 7 Real‐Time PCR System (Thermo Fisher Scientific).

Three qPCR “technical replicates” were averaged to obtain mean Ct values for each sample across each assay (Appendix 1). The limit of detection (LOD) and limit of quantification (LOQ) for each assay were evaluated using qPCRs across several DNA template concentration levels using synthetic gBlocks Gene Fragments (Integrated DNA Technologies, Coralville, Iowa, USA). LOD and LOQ were then calculated following Klymus et al. (2020). Standard curves included six (AMER and AMAU: 31,250, 6250, 1250, 250, 50, and 10 copies/reaction) or seven (AUBE: 20 million, 10 million, 5 million, 2.5 million, 1.25 million, 750,000, and 375,000 copies/reaction) serial dilutions. LOD corresponded to the lowest template concentration at which a ≥95% detection rate was observed (Bustin et al., 2009) using 24 replicate qPCRs across four (AMER and AMAU) or six (AUBE) template concentrations. LOQ corresponded to the lowest template concentration at which the coefficient of variation for Ct was ≤35% (Forootan et al., 2017) using eight replicate qPCRs across eight template concentrations (all assays).

**Figure 1:**
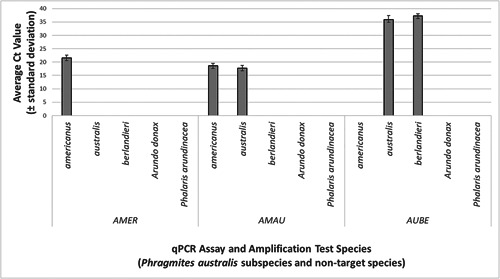
Performance of three novel qPCR assays for discriminating among *Phragmites australis* subspecies as determined by average threshold cycle (Ct) values (± standard deviation) across three technical replicates per sample (*n* = 24 for *P. australis* subsp. *americanus*, *n* = 26 for *P. australis* subsp. *australis*, *n* = 9 for *P. australis* subsp. *berlandieri*, *n* = 4 for *Arundo donax*, *n* = 4 for *Phalaris arundinacea*), with DNA normalized to 1 ng/µL for each qPCR. Lower Ct values indicate superior assay efficiency and greater likelihood of detection when DNA concentration is low. Species and subspecies for which no Ct value bar is shown did not amplify with that marker (e.g., no detection); these non‐target species/subspecies do not have a Ct value of “0”.

In our assay trials, Ct values from triplicate qPCR replicates were averaged for each sample (Appendix 1), then all sample Ct values were averaged by subspecies in order to characterize sensitivity (Figure 1). Assay sensitivity was also demonstrated by determining the LOD and LOQ for each assay (Table 3). LOD for AMER and AMAU were calculated from 24 replicates of four concentrations: 16, 8, four, and two copies/reaction. LOD for AUBE was calculated from 24 replicates of six concentrations: 2.5 million, 2 million, 1.25 million, 750,000, 500,000, and 375,000 copies/reaction. LODs for the three assays were 16 copies/reaction for AMER, eight copies/reaction for AMAU, and 2 million copies/reaction for AUBE. LOQ for AMER and AMAU were calculated from eight replicates of eight concentrations: 128, 64, 32, 16, eight, four, two, and one copies/reaction. LOQ for AUBE was calculated from eight replicates of eight concentrations: 20 million, 10 million, 5 million, 2.5 million, 2 million, 1.25 million, 750,000, and 500,000 copies/reaction. LOQs for AMER and AMAU were 128 copies/reaction and 64 copies/reaction, respectively. LOQ for AUBE was not determined, as the coefficient of variation (CV) for different concentration classes not only ranged above 35% for all classes, but also did not demonstrate the expected pattern of declining CV (i.e., increasing precision) with increasing template concentration (Klymus et al., 2020). Given the lack of that expected pattern, and a testing range that was already in the millions of copies (and thus of little practical value), further testing to determine an LOQ for AUBE was deemed unnecessary. No amplification was observed in any DNA extraction blanks or negative template controls for any assay.

**Table 3:** Efficiency of three novel *Phragmites australis* subspecies‐discriminating qPCR assays (AMER, AMAU, and AUBE), including the limit of detection (LOD) and limit of quantification (LOQ) for each, determined using synthetic gBlocks Gene Fragments (Integrated DNA Technologies) and sensitivity calculations following Klymus et al. (2020).

Parameter	AMER	AMAU	AUBE
Slope	−3.345	−3.301	−6.578
*y*‐intercept	41.106	39.037	73.437
*R* ^2^	0.957	0.99	0.757
Efficiency	99.05%	100.88%	41.91%
Error	0.177	0.082	0.932
LOD	16 copies	8 copies	2 million copies
LOQ	128 copies	128 copies	NA

*Note*: NA = not available.

## CONCLUSIONS

Three probe‐based qPCR assays were designed to genetically distinguish between three *P*. *australis* subspecies. One assay (AMER) amplifies only samples from *P. australis* subsp. *americanus*, another assay (AUBE) amplifies only samples of *P. australis* subsp. *australis* and/or *P. australis* subsp. *berlandieri*, and another assay (AMAU) amplifies only samples of *P. australis* subsp. *americanus* and/or *P. australis* subsp. *australis*. Three basic scenarios for use of these assays are described in Appendix 2. First, an unknown sample could be assayed with each assay separately until identification is reached. Second, where the introduced and Gulf Coast subspecies co‐occur, or where all three subspecies co‐occur, DNA from an individual plant could be concurrently assayed (multiplexed) with AMAU and AUBE. If the sample amplifies with both AMAU and AUBE, the sample is assigned to the introduced subspecies, *P. australis* subsp. *australis*. If the sample amplifies only with AUBE, it is assigned to the Gulf Coast subspecies, *P. australis* subsp. *berlandieri*. If the sample amplifies only with AMAU, it is assigned to the native subspecies, *P. australis* subsp. *americanus*. Where only the native and introduced subspecies co‐occur, DNA from an individual plant could be run with only the AMER assay. If the sample amplifies with AMER, it is assigned to the native subspecies, *P. australis* subsp. *americanus*, and if it does not amplify, it is assigned to the introduced subspecies, *P. australis* subsp. *australis*. The sample could also be multiplexed with AMER and AUBE, where amplification with AUBE (and not AMER) would demonstrate that the non‐amplification with AMER was not due to technical failure (e.g., DNA extraction, pipetting, or qPCR instrument error), and the sample would then be assigned with additional confidence to the introduced subspecies, *P. australis* subsp. *australis*. Because qPCR assays often perform with less efficiency (and higher LOD) when multiplexed, changes to sensitivity when multiplexing should be assessed, particularly for low‐quality samples, prior to assaying full sample sets. However, these assays were designed for the genetic discrimination of leaf tissue samples, for which the concentrations of template should, in nearly any case, be orders of magnitude above those at which efficiency and LOD would influence the outcomes.

All else being equal, including DNA template concentrations, lower Ct values indicate comparatively greater assay efficiency and greater likelihood of providing DNA detection when DNA is extremely dilute or degraded. We suspect that the performance of AUBE, in terms of sensitivity and precision (i.e., high LOD, indeterminable LOQ at high concentrations), is due to a one base pair 5ʹ–3ʹ overlap in the forward primer and probe‐binding sequence. Even with the large amount of chloroplast DNA sequence made available by our sequencing efforts, finding regions of DNA with adequate reaches of conserved and diverged sequence among the subspecies proved very difficult. The targeted regions were deemed the best options for developing the discriminatory assays, despite the primer/probe overlap and apparent interference in the AUBE assay. We note that while sensitivity and precision are critical components for qPCR assays that will be used with DNA sources that may be poor in quality and/or low in concentration (e.g., environmental DNA [eDNA]), the AMER, AMAU, and AUBE assays were not designed for such purposes. The assays instead are designed for simple genetic discrimination using DNA from DNA‐rich samples (e.g., leaf tissue), with no need for quantitative estimates of DNA template concentrations. Even very small, degraded, or small and degraded pieces of plant tissues will nearly always contain adequate concentrations of DNA template for qPCR amplification (Doyle and Doyle, 1987) with any of the assays. In such cases where one of these assays appears to not work with a suspected *Phragmites* sample, increasing the amount of DNA template in a qPCR (if too little template is suspected due to poorly preserved tissue) or, if enzymatic inhibition is suspected, diluting the DNA aliquot added to a qPCR are well‐known, easily implemented steps that often overcome such challenges.

In this study, we developed three novel qPCR assays to genetically discriminate morphologically similar subspecies of the common reed in the United States. These three *P. australis* assays are accurate (zero non‐target amplification and consistent target amplification) and offer faster identification of unknown samples than existing methods of *Phragmites* genetic discrimination (e.g., sequencing, Saltonstall, 2002; genotyping, Saltonstall 2003; RNase H–dependent PCR, Zuzak et al., 2018) to the subspecies level among all three *P. australis* subspecies present in the United States (e.g., PCR‐RFLP, Wendell et al., 2021). The specific advantages and disadvantages of each available genetic discrimination method are summarized in Table 1 of Lindsay et al. (2022). We expect these qPCR assays to be valid for the discrimination of *P. australis* subspecies across the United States, including regions of southern California and southern Arizona where all three subspecies co‐occur, because our sample set included specimens from these regions (Appendix 1). We suspect the assays to be applicable to *P. australis* subspecies in Canada and Mexico as well, although additional validation using samples from regions outside the United States would be required before implementation outside the assay design region, along with testing of any morphologically similar species (aside from *A. donax* and *P. arundinacea*) that occur in the region of new application. This step is important to ensure that the assays perform similarly on conspecific samples that may have small but impactful sequence differences within assay loci (e.g., nucleotide changes in the 3′ region of a primer resulting in substantially diminished primer‐template binding efficiency).

The three *P. australis* subspecies for which the assays were developed are of substantial ecological and infrastructure significance to the Great Lakes and Gulf Coast regions. Knowing the geographic extent of each subspecies in finer detail could lead to improved mapping capabilities and better information on the susceptibility of coastal Louisiana locales to damages from *N. biwakoensis*, making long‐term and geographically broad tracking of changes in the ranges of native and invasive *Phragmites* more feasible. Potential uses of these genetic assays include identification of resistant subspecies for use in ecosystem management and restoration along the Gulf Coast and an increased understanding of the extent of non‐native invasion and dynamics in the Great Lakes region. Initial attempts to employ these assays with field‐portable, user‐friendly technologies have proven challenging (unpublished data), but near‐future developments in rapid, portable DNA‐characterizing (e.g., qPCR) technologies should allow these and other assays to be used in the field by non‐geneticists. The emergence of these capabilities into field studies could substantially empower *Phragmites* research and management by permitting practically (if not literally) instantaneous association of a sample or stand to a particular *Phragmites* group.

## AUTHOR CONTRIBUTIONS

D.L.L. conceived the research and designed the experiments with the assistance of R.F.L. D.L.L. and X.G. performed the experiments. All authors assisted in methodology. N.E.H., J.T.C., and L.A.M. assisted with sample collection. D.L.L. acquired the funding. D.L.L. and R.F.L. analyzed the data. All authors wrote the manuscript and approved the final version of the manuscript.

## Data Availability

Data for nine chloroplast sequences (Table 1) are available online at the NCBI GenBank genetic sequence database (https://www.ncbi.nlm.nih.gov/); assigned accession numbers include: *P. australis* subsp. *americanus* (MW287631, MW287632, MW287634, MW287635), *P. australis* subsp. *australis* (MW287627, MW287628, MW287630, MW287633), and *P. australis* subsp. *berlandieri* (MW287629).

## Appendix 1

Detailed information for all samples (*N* = 67) used to design and/or test novel chloroplast genome qPCR markers to genetically discriminate *Phragmites australis* subspecies in the United States, including species/subspecies, sample/population ID, collection state (U.S.), GPS coordinates of original collection location, sample collector name, and mean Ct value ± standard deviation for each assay (n.d. = no detection). All *P. australis* samples used in this study were collected from living plants in common gardens or greenhouses and had been grown from prior field collections by *P. australis* researchers. Herbarium voucher specimens were not utilized, nor were plants from the sampled populations (some of which have since expired) archived as herbarium voucher specimens. Samples from non‐target species were field collected by local *Arundo donax* and *Phalaris arundinacea* researchers.

**Table:**

Species	Population ID	Collection state	GPS coordinates		Sample collector	Mean Ct ± SD		
			Latitude	Longitude		AMER	AMAU	AUBE
*Arundo donax* L.	Ad1‐NT	Louisiana	32.11747	−91.24467	N. Harms	n.d.	n.d.	n.d.
	Ad2‐NT	Mississippi	32.30020	−90.89871	N. Harms	n.d.	n.d.	n.d.
	Ad3‐NT	Texas	29.45860	−98.54131	A. Schad	n.d.	n.d.	n.d.
	Ad4‐NT	Texas	29.45860	−98.54131	A. Schad	n.d.	n.d.	n.d.
*Phalaris arundinacea* L.	Pa5‐NT	New York	42.70133	−78.72764	J. Unghire	n.d.	n.d.	n.d.
	Pa1‐NT	Washington	46.20417	−119.77727	J. Parsons	n.d.	n.d.	n.d.
	Pa2‐NT	Washington	42.23882	−119.22879	J. Parsons	n.d.	n.d.	n.d.
	Pa3‐NT	Washington	46.38029	−119.43216	J. Parsons	n.d.	n.d.	n.d.
*Phragmites australis* subsp. *americanus* Saltonst., P. M. Peterson & Soreng	PC‐N	California	33.82967	−116.31259	J. Cronin	23.130 ± 0.419	19.474 ± 0.279	n.d.
	SCR‐N	California	34.35527	−119.00642	J. Cronin	21.022 ± 0.524	18.101 ± 0.173	n.d.
	USGSGLSC3‐N	Indiana	41.67695	−86.99953	K. Kowalski	20.152 ± 0.409	17.958 ± 0.145	n.d.
	MEE‐N	Maine	43.87200	−69.77571	L. Meyerson	21.075 ± 0.507	17.811 ± 0.237	n.d.
	Nonesuch‐N	Maine	43.57996	−70.32811	L. Meyerson	20.260 ± 0.745	17.237 ± 0.385	n.d.
	Spurlink‐N	Maine	43.58938	−70.24548	L. Meyerson	22.879 ± 0.675	19.231 ± 0.198	n.d.
	MD‐N	Maryland	38.77000	−75.95000	J. Cronin	20.113 ± 0.208	17.653 ± 0.355	n.d.
	NIB‐N	Massachusetts	41.47249	−70.76148	L. Meyerson	21.612 ± 0.466	18.646 ± 0.308	n.d.
	*28ChelseaKowalski‐N	Michigan	42.33793	−84.00016	K. Kowalski	20.676 ± 0.238	18.104 ± 0.092	n.d.
	25SHRANWR‐N	Michigan	43.37217	−83.99883	K. Kowalski	21.642 ± 0.310	19.066 ± 0.505	n.d.
	USGSGLSC4‐N	Michigan	42.30915	−84.05627	K. Kowalski	19.871 ± 0.462	17.607 ± 0.325	n.d.
	*DUL‐N	Minnesota	46.74054	−92.06205	J. Cronin	22.556 ± 0.285	19.644 ± 0.168	n.d.
	*SC‐N	Missouri	40.06046	−95.24317	J. Cronin	20.889 ± 0.413	18.194 ± 0.108	n.d.
	PA2021USA08‐N	New York	43.07884	−76.71015	N. Harms	21.553 ± 0.774	19.188 ± 1.214	n.d.
	PA2021USA09‐N	New York	43.75157	−76.19888	N. Harms	22.246 ± 1.231	20.159 ± 0.207	n.d.
	PA2021USA14‐N	New York	44.45239	−75.74757	N. Harms	21.764 ± 0.314	19.087 ± 0.465	n.d.
	NC‐N	North Carolina	36.51000	−75.95000	J. Cronin	20.938 ± 0.319	18.140 ± 0.057	n.d.
	*CPNWR‐N	Ohio	41.67585	−83.30478	K. Kowalski	22.662 ± 0.183	19.281 ± 0.092	n.d.
	ONWRRON‐N	Ohio	41.63281	−83.23427	K. Kowalski	23.096 ± 0.322	19.677 ± 0.303	n.d.
	POR‐N	Oregon	42.75612	−124.50085	J. Cronin	21.800 ± 0.621	18.733 ± 0.226	n.d.
	15OR5‐N	Oregon	42.75676	−124.50072	L. Meyerson	23.953 ± 0.398	20.703 ± 0.140	n.d.
	USG‐N	Utah	37.09528	−113.56575	J. Cronin	20.420 ± 0.778	16.694 ± 0.327	n.d.
	ELL‐N	Washington	46.93596	−120.51472	J. Cronin	20.445 ± 0.724	17.884 ± 0.267	n.d.
	15WA7‐N	Washington	45.73225	−120.53137	L. Meyerson	22.452 ± 1.022	18.574 ± 0.333	n.d.
*Phragmites australis* (Cav.) Trin. ex Steud. subsp. *australis*	MOBI‐M	Alabama	30.66767	−87.92101	J. Cronin	n.d.	18.676 ± 0.073	37.913 ± 1.504
	I40‐M	Arizona	34.71597	−114.48858	J. Cronin	n.d.	17.872 ± 0.218	35.278 ± 0.847
	ARM1‐M	Arkansas	34.69492	−92.29224	J. Cronin	n.d.	17.477 ± 0.218	34.160 ± 4.505
	LCN‐M	California	34.54030	−119.62002	J. Cronin	n.d.	17.890 ± 0.580	35.971 ± 1.547
	WEST9‐M	California	35.49921	−120.65311	J. Cronin	n.d.	17.626 ± 0.291	35.815 ± 2.089
	CT‐M	Connecticut	41.30600	−72.35939	J. Cronin	n.d.	16.426 ± 0.344	35.128 ± 2.843
	AP‐M	Delaware	39.45000	−75.64000	J. Cronin	n.d.	17.979 ± 0.152	36.764 ± 1.852
	LN‐M	Illinois	40.22673	−89.27119	J. Cronin	n.d.	17.854 ± 0.409	35.244 ± 2.906
	*Greeny1‐M	Louisiana	29.21941	−89.30135	J. Cronin	n.d.	16.158 ± 0.207	34.276 ± 0.691
	CJ‐M	Louisiana	29.77564	−93.34022	J. Cronin	n.d.	17.368 ± 0.337	34.170 ± 2.710
	CR‐M	Louisiana	29.88000	−93.07000	J. Cronin	n.d.	16.376 ± 0.280	32.787 ± 2.350
	EC‐M	Louisiana	29.77000	−93.29000	J. Cronin	n.d.	19.221 ± 0.601	34.693 ± 2.029
	JC‐Greeny‐M	Louisiana	29.13760	−89.14510	J. Cronin	n.d.	16.994 ± 0.058	36.032 ± 0.832
	RR‐M	Louisiana	29.68000	−92.81000	J. Cronin	n.d.	17.903 ± 0.125	35.637 ± 1.023
	*DOG‐M	Louisiana	29.23722	−89.38607	J. Cronin	n.d.	16.245 ± 0.040	33.672 ± 0.330
	BUR‐M	Louisiana	29.37963	−89.60106	J. Cronin	n.d.	17.162 ± 0.204	35.620 ± 0.972
	EAR1‐M	Louisiana	29.19376	−89.29607	J. Cronin	n.d.	17.656 ± 0.488	37.544 ± 2.118
	ROD1‐M	Louisiana	29.12856	−89.28797	J. Cronin	n.d.	17.008 ± 0.234	36.067 ± 1.447
	BSC‐M	Maine	44.51000	−70.35000	J. Cronin	n.d.	16.938 ± 0.138	35.414 ± 2.323
	^TC‐M	Maryland	38.77000	−75.95000	J. Cronin	n.d.	19.010 ± 0.126	36.923
	FP‐M	Massachusetts	41.55000	−70.60000	J. Cronin	n.d.	17.998 ± 0.280	35.883 ± 0.623
	USGS‐GLSC1‐M	Michigan	42.20827	−83.55615	K. Kowalski	n.d.	18.563 ± 0.233	37.608 ± 2.001
	*14MI46‐M	Michigan	44.29368	−85.24337	L. Meyerson	n.d.	19.719 ± 0.368	38.257 ± 1.038
	USGS‐GLSC2‐M	Ohio	41.63262	−83.23419	K. Kowalski	n.d.	18.239 ± 0.288	37.504 ± 1.323
	CullenPark‐M	Ohio	41.70521	−83.47610	N. Harms	n.d.	19.072 ± 0.260	38.505 ± 0.965
	*WH‐M	Wisconsin	42.47901	−87.84930	J. Cronin	n.d.	19.219 ± 0.118	37.650 ± 0.235
*Phragmites australis* subsp. *berlandieri* (E. Fourn.) Saltonst. & Hauber	WEST4‐I	California	32.67596	−115.61351	J. Cronin	n.d.	n.d.	37.994 ± 1.969
	WEST7‐I	California	33.44666	−115.84365	J. Cronin	n.d.	n.d.	35.921 ± 2.358
	FL‐I	Florida	26.65000	−80.16000	J. Cronin	n.d.	n.d.	36.318 ± 1.729
	OB‐I	Florida	26.65939	−80.16910	J. Cronin	n.d.	n.d.	37.915 ± 1.860
	*BC‐I	Louisiana	30.05750	−90.37204	J. Cronin	n.d.	n.d.	37.260 ± 1.878
	^CR‐I	Louisiana	29.83000	−93.11000	J. Cronin	n.d.	n.d.	37.698
	IC‐I	Louisiana	29.78000	−92.20000	J. Cronin	n.d.	n.d.	37.310 ± 1.741
	PC‐I	Louisiana	29.45000	−90.46000	J. Cronin	n.d.	n.d.	38.353 ± 1.682
	ANZ‐I	Texas	26.14160	−98.32336	J. Cronin	n.d.	n.d.	38.353 ± 1.211

*Samples used for chloroplast sequencing and qPCR assay design.

^Samples that did not amplify in triplicate with assay AUBE (single amplification only).

## Appendix 2 Protocol for using chloroplast qPCR markers to genetically discriminate *Phragmites australis* subspecies.

### Materials

Materials and equipment indicated here have been used in our laboratory. Substitutions with equivalent materials can be carried out as required.

- DNA extracted from unknown sample
- Positive control (DNA extracted from confirmed *Phragmites australis* subspecies)
- Sterile, molecular biology–grade water
- TaqMan Environmental Master Mix 2.0 (Thermo Fisher Scientific, Waltham, Massachusetts, USA)
- Primers and probes for AMER, AMAU, and AUBE assays (see Table 2)
- Gloves, pipettes, pipette tips, 1.5–2.0 mL Eppendorf tubes, 384‐well sample plate
- Quantitative real‐time PCR machine (Applied Biosystems ViiA 7 Real‐Time PCR System, Thermo Fisher Scientific).

### Procedure

1. DNA from samples of *P. australis* subsp. *americanus* amplifies with assays AMER and AMAU. DNA from samples of *P. australis* subsp. *australis* amplifies with assays AMAU and AUBE. DNA from samples of *P. australis* subsp. *berlandieri* amplifies with assay AUBE only. DNA from an unknown plant in an area where all three subspecies co‐occur should be assayed with AMAU and AUBE, at minimum. DNA samples from an unknown plant where only *P. australis* subsp. *americanus* and *P. australis* subsp. *australis* co‐occur can be assayed with AMER only, or with AMER and AMAU.
2. Generate the reaction mix: Combine 1X TaqMan Environmental Master Mix 2.0, 0.5 µM of each primer (forward and reverse), 0.125 µM of each probe, and 1 µL of DNA template (quantity may range from 1–20 ng/µL) in a 20 µL volume for each sample. Assay each sample in triplicate and include both a positive (confirmed *P. australis* DNA) and negative (sterile, molecular‐grade water) control (each also run in triplicate). Samples can be run across each assay singly or combined, although multiplexing may require further optimization or additional replicates to ensure accuracy. Generate as much master mix solution as necessary for the number of samples and replicates you will assay.
3. Pipette 19 µL of reaction mix into each well of a 384‐well plate, then pipette 1 µL of sample DNA (or water) into each well.
4. Set the Quantitative Real‐Time PCR Machine for a standard run with temperature cycling including an initial denaturation step at 95°C for 10 min, followed by 40 cycles of 95°C for 15 s and 60°C for 1 min. Assign sample names to each well and run.
5. Review the results of the amplification plots and Ct values for amplification or non‐amplification across each assay to determine identity. If the sample amplifies with both AMAU and AUBE, it is assigned as *P. australis* subsp. *australis*, if the sample amplifies only with AUBE it is assigned as *P. australis* subsp. *berlandieri*, and if the sample amplifies only with AMAU and/or AMER it is assigned as *P. australis* subsp. *americanus*.
6. Robust and highly reliable results are those wherein the same outcome is observed for all three replicate qPCRs. When all replicates are not in agreement, a second round of replicate assays may be required to increase confidence in identification. If inconsistent results persist or more than one assay exhibits inconsistent results, then a challenging DNA sample is indicated. Extracted DNA solutions from old, degraded, or small (weight) samples that exhibit inconsistent outcomes may suffer from low concentrations of intact DNA template, and consistent results might be achieved by increasing the solution volume (and concentration of DNA) included in the qPCR. DNA solutions from fresh or large samples that exhibit inconsistent outcomes may suffer from PCR inhibition, and consistent results might be achieved by decreasing the solution volume (and concentration of inhibitors) included in the qPCR. Unrepeatable positive results for an assay in one or more samples, or intermittent positive results across multiple samples, are indicative of potential contamination from DNA exogenous to the sample DNA extract (e.g., PCR amplicons on equipment or in reaction‐consumable stocks).
